# Comparison of signaling interactions determining annual and perennial plant growth in response to low temperature

**DOI:** 10.3389/fpls.2014.00794

**Published:** 2015-01-12

**Authors:** Astrid Wingler

**Affiliations:** Research Department of Genetics, Evolution and Environment, University College LondonLondon, UK

**Keywords:** cold acclimation, gibberellin, jasmonate, perennial ryegrass (*Lolium perenne*), poplar (*Populus*), sink limitation, sugar signaling, trehalose-6-phosphate

## Abstract

Low temperature inhibits plant growth despite the fact that considerable rates of photosynthetic activity can be maintained. Instead of lower rates of photosynthesis, active inhibition of cell division and expansion is primarily responsible for reduced growth. This results in sink limitation and enables plants to accumulate carbohydrates that act as compatible solutes or are stored throughout the winter to enable re-growth in spring. Regulation of growth in response to temperature therefore requires coordination with carbon metabolism, e.g., via the signaling metabolite trehalose-6-phosphate. The phytohormones gibberellin (GA) and jasmonate (JA) play an important role in regulating growth in response to temperature. Growth restriction at low temperature is mainly mediated by DELLA proteins, whose degradation is promoted by GA. For annual plants, it has been shown that the GA/DELLA pathway interacts with JA signaling and C-repeat binding factor dependent cold acclimation, but these interactions have not been explored in detail for perennials. Growth regulation in response to seasonal factors is, however, particularly important in perennials, especially at high latitudes. In autumn, growth cessation in trees is caused by shortening of the daylength in interaction with phytohormone signaling. In perennial grasses seasonal differences in the sensitivity to GA may enable enhanced growth in spring. This review provides an overview of the signaling interactions that determine plant growth at low temperature and highlights gaps in our knowledge, especially concerning the seasonality of signaling responses in perennial plants.

## INTRODUCTION

Reduced plant growth at low temperature affects the productivity of agricultural as well as natural ecosystems. In addition, growth restriction determines the geographical distribution of plants. While cold extremes during the winter may affect survival, reduced growth at low temperature during the growing season is a key factor limiting plant distribution globally. For example, the treeline is globally determined by the length and average temperature of the growing season (Paulsen and Körner, 2014). The low temperature limit at which plants can grow is about 5°C, independent of the growth form, life cycle or geographic location, despite the fact that photosynthesis can continue well below this temperature (Körner, 2008).

Reduced growth at low temperature is not only a negative consequence of slowed down metabolism, but an active process that may be important for survival. There is a trade-off between allocation of resources, especially carbon, into growth and into pathways for cold acclimation. This requires regulatory processes, principally mediated by phytohormones that control growth dependent on the environmental conditions. In turn, this regulation results in altered carbon dynamics and generates sugar signals that further modulate metabolic pathways involved in biosynthetic and catabolic processes.

Plant organ growth is determined by cell division and cell expansion. Cell division depends on the activity of the mitotic cell cycle, while cell expansion is a complex process that can involve endoreduplication of the genome without cell division and turgor-driven growth combined with cell wall loosening and synthesis of cell wall material. In leaves, early stages of growth are determined by carbon supply, whereas later in development hydraulic, turgor-driven factors may constrain growth (Pantin et al., 2011). During stress conditions, both cell division and expansion can be impaired (Skirycz and Inzé, 2010) in a process that is actively regulated by phytohormone signaling pathways (Claeys et al., 2014; Wasternack, 2014). However, while low temperature conditions often result in dwarf phenotypes, this may mainly be due to reduced elongation growth, which not always results in lower total shoot biomass, as shown for cold-acclimated winter cereals (Hüner et al., 2014).

Signaling pathways restricting growth, and interactions between them, have so far predominantly been explored for *Arabidopsis*, but research on perennial models, such as grasses and poplar species, suggests that the main signaling components are conserved. However, their importance, especially in a seasonal context, may differ between annuals and perennials that need to survive the winter and resume growth in spring. Annual growth cycles require sophisticated mechanisms that allocate resources into growth, storage or protective pathways, dependent on current and impending environmental conditions. This review therefore includes fundamental findings from research on annual models, such as *Arabidopsis* and compares these findings with our knowledge of molecular regulatory mechanisms in perennials (herbaceous and woody). While covering some of the regulatory processes that result in growth cessation in autumn, this review does not specifically address the control of bud dormancy in response to temperature. Excellent reviews on the signaling pathways that control dormancy have been published elsewhere (e.g., Olsen, 2010; Cooke et al., 2012).

## LOW TEMPERATURE RESULTS IN SINK LIMITATION

Although it is often assumed that plant growth is determined by rates of photosynthesis, this is not necessarily the case. While low temperature reduces Calvin cycle activity, cold acclimation can overcome this limitation (Holaday et al., 1992; Hurry et al., 1995). For example, in winter crops cold acclimation enhances sink activity by increased sucrose synthesis which leads to increased recycling of phosphate and regeneration of ribulose bisphosphate (Kurepin et al., 2013). Substantial rates of photosynthesis can therefore be maintained. In alpine plants, photosynthesis is still active at very low temperatures: the evergreen perennial *Saxifraga paniculata* only shows a reduction in photosynthesis at temperatures below 5°C, and considerable photosynthetic activity still occurs at temperatures well below zero (Hacker and Neuner, 2006). Even in temperate pastureland, photosynthesis continues at temperatures as low as -4°C (Skinner, 2007). Despite this, significant growth only occurs at temperatures above 5°C, which is in agreement with limiting growth temperatures in winter crops and at the treeline (Körner, 2008).

Growth is also affected more severely than photosynthesis during moderate water deficit, resulting in increased carbon availability (Muller et al., 2011). This shows that plant growth during stress can be sink- and not source-limited. Sink limitation can result in the accumulation of carbohydrates in the form of sugars, which, in turn, suppress photosynthesis via feedback inhibition of photosynthetic gene expression (e.g., Rolland et al., 2002). Thus, the accumulation of sugars that typically occurs at low temperature could theoretically result in suppression in photosynthetic genes. However, in winter crops feedback inhibition of photosynthesis is overcome and photosynthetic capacity can even be increased (Hüner et al., 2014). Similarly, photosynthesis in *Arabidopsis* can partially recover during cold acclimation (Savitch et al., 2001) and leaves of plants that developed at low temperature do not show sugar-dependent feedback inhibition (Strand et al., 1997). This effect of cold acclimation does not only allow plants to sustain high rates of photosynthesis while also achieving enhanced freezing tolerance, but also has consequences for plant development. In aging leaves, sugar accumulation can down-regulate photosynthetic gene expression and accelerate leaf senescence (Pourtau et al., 2006; Wingler et al., 2009), but this response is abolished by cold acclimation (Masclaux-Daubresse et al., 2007), resulting in longer photosynthetic lifespan. While these interactions have mainly been investigated in annual plants, mainly *Arabidopsis*, our recent research suggests that such interactions between sugar and temperature signaling also occur in the perennial *Arabis alpina* (Wingler et al., 2012, 2014).

In contrast to winter crops and *Arabidopsis*, photosynthesis is strongly down-regulated during winter in evergreen conifers (Adams et al., 2002; Öquist and Huner, 2003). However, as low temperature restricts root as well as shoot growth, non-structural carbohydrates can accumulate nevertheless, as e.g., shown for *Pinus mugo* (Hoch and Körner, 2009). In addition, it was shown that metabolizable carbon reserves increase in evergreen and deciduous trees with altitude (Hoch and Körner, 2003; Shi et al., 2008; Molina-Montenegro et al., 2012). This work supports the view that in perennials plants low temperature affects meristematic growth processes at the cellular level more severely than photosynthesis, resulting in sink limitation instead of source limitation.

## CARBOHYDRATE METABOLISM AND CELL WALL BIOSYNTHESIS IN RESPONSE TO LOW TEMPERATURE

Cold acclimation requires adjustment of carbon allocation to different pools. For *Arabidopsis* it was shown that starch content may initially increase upon transfer to low temperature until growth is partially restored after which starch content declines (Gorsuch et al., 2010). This degradation of starch supports cold tolerance by allowing accumulation of sugars and other compatible solutes (Kaplan and Guy, 2005; Yano et al., 2005; Li et al., 2011). Transfer to low temperature also results in increased activity of the sucrose biosynthetic enzyme sucrose phosphate synthase (SPS; Strand et al., 1999) and overexpression of SPS improves freezing tolerance (Strand et al., 2003).

Similar changes in carbohydrate metabolism also occur in perennials. Overall, non-structural carbohydrates accumulate universally during cold acclimation in different plant functional groups, including perennials (Campbell et al., 2007). For example, soluble sugars (such as glucose, fructose, sucrose, raffinose, and trehalose) accumulate in the leaves of deciduous trees in response to chilling (Renaut et al., 2004). In the leaves of evergreen trees, gymnosperms as well as angiosperms, starch content declines during the winter and during cold hardening, but soluble sugar content increases (Tinus et al., 2000; Reyez-Díaz et al., 2005).

While starch is the dominant form of stored carbon in the majority of plants, other species, in particular grasses, can also store carbon in the form of fructans. Enhanced fructan synthesis in cold-acclimated winter wheat may overcome feedback limitation of photosynthesis (Savitch et al., 2000). In perennial grasses, fructans accumulate in response to low temperature in autumn and can be used for growth in spring (e.g., Pollock and Jones, 1979; Tamura et al., 2014). In addition to this storage role, fructans also have a protective function during stress (Sandve et al., 2011; Van den Ende, 2013). Furthermore, it has been suggested that raffinose family oligosaccharides and small fructans can serve as phloem-mobile signals (Van den Ende, 2013). In this context, the fructan-degrading enzymes, fructan exohydrolases, could play an important role in generating stress signals, even in non-fructan plants (Van den Ende et al., 2004).

At the cellular level, growth involves the synthesis of cell wall material. Sucrose is not only a compatible solute, it is also the main transport sugar in plants and required for cellulose biosynthesis during the formation of the cell wall. It can be degraded by sucrose synthase into fructose and UDP-glucose, which is a direct substrate for cellulose synthases. In cotton, flux from glucose to sucrose for cellulose synthesis, appears to be reduced at low temperature (Martin and Haigler, 2004). Overexpression of sucrose synthase in cotton can increase cellulose production, but does not affect growth (Coleman et al., 2009), neither does overexpression of SPS (with the aim of improving sucrose availability for cell wall biosynthesis) affect growth of poplar, although impacts were found on phenology (Park et al., 2009). This shows that there is not a clear direct relationship between sucrose synthesis, cellulose biosynthesis, and growth. In addition to providing the components for cell wall biosynthesis, carbon metabolism generates the turgor pressure which, together with cell wall loosening by expansins (Cosgrove et al., 2002), drives cell expansion. Under stress, cell wall extensibility can however, be reduced. For example in cotton, fiber elongation is reduced at low temperature, although the effect on expansin protein abundance is cultivar dependent (Zheng et al., 2012).

Overall, plants are conservative in their use of carbon at low temperature and maintain carbohydrate stores rather than investing their resources into growth-related processes, such as cell wall biosynthesis. This may be particularly important in perennials at high latitudes as they experience short days in combination with low light intensity in winter, which may have a more severe effect on photosynthetic carbon fixation than the low temperature as such.

## SIGNALING PATHWAYS CONTROLLING GROWTH IN RESPONSE TO LOW TEMPERATURE

Conservative use of resources at low temperature enables perennials to maintain sufficient carbon reserves for resumption of growth in warmer conditions in spring (see Enhanced Growth After the Relief of Sink Limitation). Such seasonal adjustment requires active regulatory processes that limit growth at low temperature and promote growth at warm temperature, beyond the direct effects on temperature on metabolic rate.

The initial events in cold perception in plants are not entirely resolved, but they include Ca^2+^ influx into the cell, probably triggered by changes in membrane fluidity and mediated by the cytoskeleton (Knight and Knight, 2012). Stablilization of inducer of CBF expression (ICE) protein then induces the expression of C-repeat binding factor (CBF) genes, which trigger transcriptional changes that are required for cold acclimation (Sharma et al., 2005; Knight and Knight, 2012). Although a lot of the work on cold acclimation has been carried out in *Arabidopsis*, the CBF pathway has also been shown to be active in perennial plants, such as perennial ryegrass (Xiong and Fei, 2006), poplar (Benedict et al., 2006), and birch (Welling and Palva, 2008).

In addition to improving cold tolerance, overexpression of *CBF* genes inhibits growth in *Arabidopsis* (Liu et al., 1998; Gilmour et al., 2000) and perennials (Benedict et al., 2006; Xiong and Fei, 2006). Important interactions have been shown between phytohormones and the CBF pathway, in particular jasmonates (JAs) as upstream regulators (see Antagonistic Effects of Jasmonate and Gibberellins on Growth) and the gibberellin (GA)/DELLA pathway regulating growth downstream of CBFs (see Growth Inhibition by the CBF Pathway is Mediated by GA/DELLA), in addition to interactions between JA and GA signaling (see Antagonistic Effects of Jasmonate and Gibberellins on Growth). The role of abscisic acid (ABA) is less clear as it can inhibit as well as activate growth, but a role of ABA in growth cessation in autumn has been proposed (see A Role for Abscisic Acid in Growth Cessation in Autumn?). There are also important interactions between daylength and the hormone signaling pathways, which could potentially explain seasonality of the growth response in anticipation of a change in temperature (see Interactions with Light Conditions and Daylength).

### THE FUNCTION OF GIBBERELLINS AND INHIBITION OF GROWTH BY DELLA PROTEINS

Probably the best-studied pathway that underlies control of growth at low temperature is the GA/DELLA pathway. The active GAs GA1 and GA4 are phytohormones stimulating cell division and expansion (Claeys et al., 2014), whereas DELLA proteins are growth repressors belonging to the GRAS family of transcriptional regulators. Their function has been studied in detail in *Arabidopsis*: in the presence of GA DELLAs are degraded via ubiquitination and the 26S proteasome pathway, thus overcoming the restraint of growth by DELLAs (Achard and Genschik, 2009; Colebrook et al., 2014; Xu et al., 2014). Cold stress decreases the contents of bioactive GAs which results in accumulation of DELLAs and inhibition of plant growth (Achard et al., 2008). In addition to restraining growth, DELLAs also increase survival during freezing in *Arabidopsis* (Achard et al., 2008). While action of DELLAs generally results in dwarfing, this may mainly be due to reduced stem elongation while overall biomass may not be affected, as shown for cold-acclimated winter cereals (Hüner et al., 2014).

There is evidence that the GA/DELLA pathway is also active in perennials, for example, in poplar. Although this has not specifically been analyzed with respect to the cold-dependent restriction of growth, it was demonstrated that GA-deficient or -insensitive poplar plants exhibit various degrees of dwarfism (Zawaski et al., 2011). Moreover, shoot growth in transgenic plants with increased DELLA expression was reduced (Busov et al., 2006) and hypersensitive to drought and short days (Zawaski and Busov, 2014).

In addition to the general role of the GA/DELLA pathway in regulating growth in response to temperature, reduced GA content is also responsible for growth cessation in autumn as part of the dormancy cycle of perennials (Olsen, 2010; Cooke et al., 2012). While shortening of the photoperiod is an important signal resulting in lower rates of GA synthesis in trees in autumn (Eriksson and Moritz, 2002; Mølmann et al., 2003), low night temperatures in combination with inhibition of GA synthesis can overcome the requirement for short days in growth cessation (Mølmann et al., 2005). Interactions with daylength are further discussed in Section “Interactions with Light Conditions and Daylength.”

### GROWTH INHIBITION BY THE CBF PATHWAY IS MEDIATED BY GA/DELLA

In annual plants, overexpression of *CBF* genes inhibits growth (Liu et al., 1998; Gilmour et al., 2000). DELLA proteins are at least partially responsible for the inhibition of growth by the CBF pathway. For *Arabidopsis* this is supported by the finding that constitutive overexpression of the *CBF1* gene results in lower GA content and in DELLA accumulation through enhanced DELLA gene expression and protein stability (Achard et al., 2008). In a DELLA double mutant, the dwarfism caused by *AtCBF1* expression is suppressed, demonstrating a role of DELLAs downstream of the CBF pathway. Expression of a *CBF* gene from the cold-tolerant annual *Capsella bursa-pastoris* in tobacco results in dwarfism due to delayed cell division, in addition to reduced GA content (Zhou et al., 2014). GA treatment was shown to reduce *CBF* expression in *C. bursa-pastoris* to some extent, indicating that there could also be a role of GA upstream of the CBF pathway in addition to its downstream effects. The inhibition GA-dependent elongation growth by the CBF pathway is also discussed for winter cereals by Kurepin et al. (2013).

Experiments expressing *CBF* genes from *Arabidopsis* in other species and vice versa show that CBF proteins also restrict growth in perennials: for example, constitutive overexpression of *CBF* genes from perennial ryegrass (Xiong and Fei, 2006) or birch (Welling and Palva, 2008) in *Arabidopsis* resulted in stunted growth. It was also shown that expression of the *Arabidopsis AtCBF1* gene in poplar does not only lead to improved freezing tolerance, but also inhibits growth during propagation on agar, although growth recovered upon transfer to soil (Benedict et al., 2006). Given the conserved function of the CBF-dependent cold acclimation pathway and its effect on growth, it is likely that GA and DELLA mediate the growth response to changes in *CBF* expression in perennial plants too, but this remains to be shown experimentally.

### ANTAGONISTIC EFFECTS OF JASMONATE AND GIBBERELLINS ON GROWTH

Jasmonate is a hormone involved in the response to stress and has a negative effect on growth (Wasternack, 2014). Recent research with *Arabidopsis* has shown that JA reduces cell number and size by inhibiting the mitotic cell cycle as well as the endoreduplication cycle associated with cell expansion (Noir et al., 2013). Treatment with the JA agonist coronatine results in a rapid reduction in growth and photosynthetic gene expression, but photosynthetic activity is maintained (Attaran et al., 2014). This shows that, as during cold stress, the primary reason for reduced growth cannot be explained with lower rates of photosynthesis in response to JA. While the effect of JA on growth has mainly been discussed in terms of a trade-off between defense and growth, the growth restriction by JA may also be important during abiotic stress in order to divert carbon into sugars for cold acclimation. GA and JA have antagonistic effects on growth. JA can inhibit the synthesis of active GAs (Heinrich et al., 2013) and there is also interaction of the signaling pathways via DELLA proteins: in *Arabidopsis*, JA was shown to induce expression of the DELLA gene *RGL3*, which, in turn, is involved in JA signaling (Wild et al., 2012). DELLAs modulate JA signaling by interacting with the JAZ repressors of JA signaling (Song et al., 2014; Xu et al., 2014). Furthermore, *CBF* genes are upregulated by JA in *Arabidopsis* (Hu et al., 2013), as well as in the cold-tolerant annual plant *C. bursa-pastoris* (Zhou et al., 2014). This can restrain growth further via the negative effect of CBF on GA content and the positive effect on DELLA protein expression and stability (see Growth Inhibition by the CBF Pathway is Mediated by GA/DELLA). The antagonistic interactions between JA and GA may thus be direct or mediated by the CBF pathway.

It is likely that JA interactions with GA signaling also play a role in the growth response to temperature in perennial plants. For example, JA content increased in response to cold treatment in the perennial *A. alpina*, but contents of the active GAs GA1 and GA4 remained largely unchanged (Wingler et al., 2014).

### A ROLE FOR ABSCISIC ACID IN GROWTH CESSATION IN AUTUMN?

Abscisic acid is commonly considered to inhibit growth under stress conditions, in particular during drought. However, its effects on plant growth are controversial with positive and negative growth effects reported (Skirycz and Inzé, 2010; Tardieu et al., 2010). In poplar, e.g., a positive relationship was found between cambial growth and ABA content, in addition to a positive effect of external ABA application on cambial activity (Arend and Fromm, 2013). Overall, the function of ABA is complex: apart from having non-hydraulic effects, ABA induces stomatal closure and improves water conductance, which in combination can promote or inhibit growth dependent on the conditions (Tardieu et al., 2010).

Despite these contrasting effects on growth, the involvement of ABA in the response to dehydration is well-established, whereas its function in cold response is less well-defined (Knight and Knight, 2012). There may be roles of ABA in the CBF-dependent and -independent regulation of gene expression and a function of ABA in dehydration caused by freezing (Sharma et al., 2005). Overall, it is however, not obvious whether or not ABA plays a major role in inhibiting growth in response to cold stress.

A more specific role of ABA has been proposed for the temperature- and photoperiod-dependent growth cessation in trees in autumn. Although ABA treatment does not generally result in growth cessation and dormancy, there are interesting interactions with photoperiod that may determine the function of ABA in the seasonal growth cycle. In poplar buds, cold treatment leads to a transient increase in ABA under short day conditions (Welling et al., 2002). In addition, short day treatment on its own increases ABA content transiently in birch and poplar (Rinne et al., 1998; Rohde et al., 2002) and this increase is related to freezing tolerance (Welling et al., 1997; Rinne et al., 1998). On the other hand, high endogenous ABA or ABA treatment under long-day conditions did not induce growth cessation (Welling et al., 1997), suggesting that the short day dependent increase in ABA is not responsible for bud growth cessation. The role of ABA in the process therefore remains unclear (Olsen, 2010; Cooke et al., 2012).

### INTERACTIONS WITH LIGHT CONDITIONS AND DAYLENGTH

For perennial plants of high latitudes, low temperatures are often associated with short days and also low light intensity. This has a direct impact on photosynthesis that is probably more severe than that of low temperature itself and may require growth restriction to conserve carbon resources. In addition, shortening of the photoperiod is an important signal allowing plants to anticipate lower temperatures (Eriksson and Webb, 2011), e.g., before damage by sudden frost can occur. It is therefore not surprising that light signaling plays a role in the cold acclimation response. Both a low red/far red ratio and mutation in phytochrome B can increase *CBF* gene expression and freezing tolerance (Franklin and Whitelam, 2007; Lee and Thomashow, 2012). In *Arabidopsis*, *CBF* gene expression is under circadian control and increases under short days (Lee and Thomashow, 2012), while short day treatment has been shown to contribute to increased freezing tolerance in the perennial *Boechera stricta* (Heo et al., 2014). In addition, when cold stress is combined with high light intensity, reduced electron consumption by the Calvin cycle alters the redox state of the photosystem II electron acceptor plastoquinone, thus creating increased excitation pressure. It was proposed that this can regulate the expression of *CBF* genes via retrograde signaling and thereby lead to reduced elongation growth (Kurepin et al., 2013; Hüner et al., 2014).

Photoperiod also plays an important role in phytohormone synthesis and signaling, as discussed for GA/DELLA (see The Function of Gibberellins and Inhibition of Growth by DELLA Proteins) and ABA (see A Role for Abscisic Acid in Growth Cessation in Autumn?). In addition to reduced GA synthesis in response to short photoperiod in trees (Eriksson and Moritz, 2002; Mølmann et al., 2003), its synthesis can be stimulated by long photoperiods in perennial ryegrass (MacMillan et al., 2005). In particular the impact of increased *CBF* expression under short days on DELLA-dependent growth inhibition has the potential to restrict growth in perennials in autumn. However, these interactions have so far only been found in the annual species *Arabidopsis* (Achard et al., 2008) and *C. bursa-pastoris* (Zhou et al., 2014). GA signaling in *Arabidopsis* is also gated by the circadian clock resulting in increased DELLA stability during the day and increased GA sensitivity during the night (Arana et al., 2011). This regulation controls rhythmic growth during *Arabidopsis* hypocotyl elongation. Whether or not daylength-dependent regulation also underlies seasonality in the growth response to temperature in perennial plants is discussed in Section “Mechanisms Stimulating the Growth of Perennial Grasses in Spring.”

## ENHANCED GROWTH AFTER THE RELIEF OF SINK LIMITATION

Growth can be stimulated once the restraint of low temperature on sink activity is relieved. Stored carbon reserves fuel this growth before photosynthetic rates increase in the new leaves in spring.

### MECHANISMS STIMULATING THE GROWTH OF PERENNIAL GRASSES IN SPRING

The growth of perennial grasses is typically strongest early in the growing season, resulting in a transient growth spurt in spring (Peacock, 1975; Parsons and Robson, 1980; Davies et al., 1989). A decline of fructans during active growth in spring (Pollock and Jones, 1979) suggests that early season growth is fuelled by fructan degradation. Overall, Davies et al. (1989) found a negative relationship between soluble carbohydrate content and growth upon transfer to warm temperature, indicating that growth is determined by carbohydrate utilization (i.e., sink activity). Enhanced growth of perennial grasses is not solely dependent on increased temperature, but varies according to which time of year plants are exposed to warm conditions. Leaf extension rates were higher when plants were transferred to warmer temperature in March than earlier or later in the season (Davies et al., 1989). In addition, moderate frost treatment can stimulate compensatory growth to a greater extent earlier in the year (Østrem et al., 2010).

The growth response in grasses to temperature is regulated by GA. Growth of perennial ryegrass increases after GA application, even without nitrogen addition, but a positive interaction was found with nitrogen supply (Ball et al., 2012; Parsons et al., 2013). Importantly, plants collected from a pasture in winter showed a stronger response to GA than plants collected in summer (Ball et al., 2012), demonstrating that seasonal factors determine the capacity for growth. What these seasonal factors that determine GA response may be remains unresolved. GA synthesis in perennial ryegrass is stimulated by long-day treatment and GA can replace the requirement for long-days to induce flowering independent of vernalization (MacMillan et al., 2005). A likely candidate for the seasonality of the GA response would thus be photoperiod (see The Function of Gibberellins and Inhibition of Growth by DELLA Proteins and Interactions with Light Conditions and Daylength), but Parsons et al. (2013) found that photoperiod did not interact with the GA response. This suggests that the seasonal effect may instead be determined by developmental factors (Peacock, 1975; Ball et al., 2012). These factors could be epigenetic and may include floral induction by vernalization. Vernalized plants, e.g., showed higher rates of leaf extension than non-vernalized plants (Stapleton and Jones, 1987). In *Arabidopsis*, *CBF* expression delays flowering, but floral induction after vernalization results in the repression of *CBF* genes via *SOC1* (Seo et al., 2009; Wingler, 2011). This feedback loop could overcome the growth-inhibiting effect of CBF proteins in spring. However, short-term exposure to low temperature later in the season also stimulates growth of vegetative tillers, suggesting that floral induction is not required for enhanced growth of grasses (Davies et al., 1989).

### TREHALOSE-6-PHOSPHATE AS A SIGNAL TO STIMULATE PLANT GROWTH AFTER RELIEF OF SINK LIMITATION

While fructan degradation fuels growth in grasses, starch has a similar role in plants that do not accumulate fructans. In a study with 94 *Arabidopsis* accessions, it was found that biomass was negatively correlated with starch content showing that starch utilization and not its storage is responsible for growth (Sulpice et al., 2009). An important signal that controls carbohydrate metabolism is trehalose-6-phosphate (T6P). Recently, it was demonstrated that it inhibits starch degradation (Martins et al., 2013). Since T6P content reflects sucrose availability (Nunes et al., 2013a; Yadav et al., 2014), T6P can serve as a signal for low sink activity resulting in inhibition of starch breakdown into sugars during the night and preventing exhaustion of carbohydrate reserves under unfavorable conditions such as low temperature (Martins et al., 2013).

Models have been proposed that explain the role of T6P in promoting growth dependent on carbon availability (Schluepmann et al., 2012; O’Hara et al., 2013). This signaling role of T6P is important for the regulation of growth in response to changes in sink strength that occur under stress conditions. Under stress that results in sink limitation, such as cold stress, sucrose and T6P contents increase in parallel in *Arabidopsis* (Nunes et al., 2013a), although it is not clear how the change in T6P content in response low temperature is regulated at the molecular level (Nunes et al., 2013b). By inducing biosynthetic genes, including those for protein and cell wall biosynthesis (Zhang et al., 2009; Paul et al., 2010), T6P can prime plants for growth (Nunes et al., 2013a). Once sink limitation is released upon transfer from cold to warm temperatures, active biosynthetic processes enable enhanced growth. This effect is probably mediated by inhibition of the SNF-related protein kinase SnRK1 by T6P (Zhang et al., 2009). Similar regulatory roles of T6P have been confirmed for other species, including wheat (Martínez-Barajas et al., 2011) and potato (Debast et al., 2011), but not much is known about its function in perennials. It is possible that T6P-dependent growth stimulation is involved in the growth spurt of perennial grasses in spring, e.g., via an effect on fructan metabolism. Trehalose feeding to barley leaves stimulates fructan biosynthesis (Müller et al., 2000), but if this effect is mediated by trehalose itself or by T6P (which accumulates in response to trehalose supply) is unknown.

## SINK LIMITATION MODEL FOR GROWTH AT LOW TEMPERATURE

**FIGURE 1:**
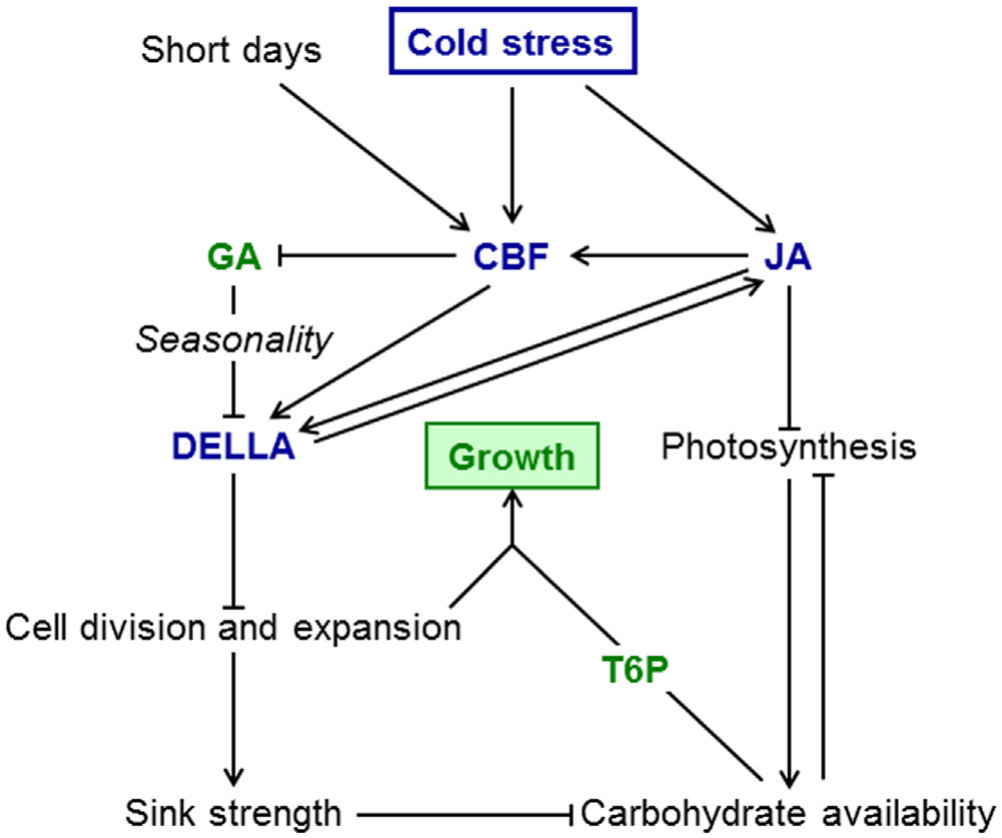
**Sink limitation model for growth restriction at low temperature.** Growth-promoting factors are in green, growth-inhibiting factors in blue. Cold stress results in induction of the CBF pathway and JA accumulation. JA inhibits photosynthesis and also induces *CBF* genes further. Interactions between JA and DELLA may be direct or indirect via the CBF pathway. The CBF pathway results in lower GA thus increasing DELLA stability. DELLA proteins inhibit cellular growth and therefore sink strength. At low temperature, reduced sink strength leads to accumulation of carbohydrates, in particular sugars. When temperature increases in spring and the restriction of growth by DELLA proteins is released, cellular growth resumes and sink limitation is overcome. Carbohydrates stored in winter (including fructans) provide substrates for biosynthetic pathways. In annual plants T6P signals the availability of carbohydrates for biosynthetic processes, but its role in the growth of perennials is still unknown. In addition to temperature, seasonality of GA and T6P sensitivity may contribute to a growth spurt in spring. Photoperiod effects, e.g., on *CBF* expression, could potentially contribute to seasonality of growth, but seasonality in the response of perennial grasses to GA is independent of photoperiod.

Although the ability restore photosynthesis during cold acclimation is species dependent (Öquist and Huner, 2003), most plants maintain considerable rates of photosynthesis at temperatures that inhibit growth almost completely. However, low light intensities and short days may result in reduced photosynthetic carbon fixation, especially at high latitudes. To maintain adequate carbon supply to achieve frost tolerance through accumulation of solutes and to provide carbon for re-growth when temperature increases, an active mechanism to restrain growth at low temperature is required. A model summarizing the signaling interactions that restrict growth at low temperature is shown in **Figure 1**. Given the changes in temperature perennial plants encounter throughout the year, the pathways that allow them not only to survive the winter, but importantly also regulate growth during the growing season may play an even more important role than in annual plants. Some aspects, in particular interactions with daylength, have been researched extensively in perennials, mainly because of the interest in the regulation of dormancy in trees. For other signaling interactions we only have information for annual models, e.g., for the interactions between the JA, GA/DELLA, and CBF pathways in regulating growth in response to temperature. However, there is sufficient evidence that the pathways themselves are conserved and active in perennial plants. More research is required to analyze the importance of interactions between these signaling pathways in the seasonal context.

If and how T6P regulates growth in perennials, especially upon relief of sink limitation in spring, is another interesting question that should stimulate research. Intriguing are also seasonal factors that, e.g., underlie the differential growth response to GA dependent on the time of year. Daylength may be able to explain some aspects of seasonality, but was excluded as a basis of altered GA sensitivity. Epigenetic changes related to vernalization during the winter may play an important role in the growth response to temperature, thus preventing plants from growing too early in the year in response to warm spells. Such mechanisms are important to prevent premature exhaustion of carbohydrates and a lack of compatible solutes which are required to protect the plants should they encounter sudden frosts.

## Conflict of Interest Statement

The author declares that the research was conducted in the absence of any commercial or financial relationships that could be construed as a potential conflict of interest.

## References

[B1] Achard P. Genschik P. (2009). Releasing the brakes of plant growth: how GAs shutdown DELLA proteins. *J. Exp. Bot.* 60 1085–1092 10.1093/jxb/ern301 19043067

[B2] Achard P. Gong F. Cheminant S. Alioua M. Hedden P. Genschik P. (2008). The cold-inducible CBF1 factor-dependent signaling pathway modulates the accumulation of the growth-repressing DELLA proteins via its effect on gibberellin metabolism. *Plant Cell* 20 2117–2129 10.1105/tpc.108.058941 18757556 PMC2553604

[B3] Adams W. W. Demmig-Adams B. Rosenstiel T. N. Brightwell A. K. Ebbert V. (2002). Photosynthesis and photoprotection in overwintering plants. *Plant Biol.* 4 545–557 10.1055/s-2002-35434

[B4] Arana M. V. Marín-de la Rosa N. Maloof J. N. Blázquez M. A. Alabadi D. (2011). Circadian oscillation of gibberellin signaling in *Arabidopsis*. *Proc. Natl. Acad. Sci. U.S.A.* 108 9292–9297 10.1073/pnas.1101050108 21576475 PMC3107313

[B5] Arend M. Fromm J. (2013). Concomitant analysis of cambial abscisic acid and cambial growth activity in poplar. *Trees* 27 1271–1276 10.1007/s00468-013-0875-z

[B6] Attaran E. Major I. T. Cruz J. A. Rosa B. A. Koo A. J. K. Chen J. (2014). Temporal dynamics of growth and photosynthesis suppression in response to jasmonate signaling. *Plant Physiol.* 165 1302–1314 10.1104/pp.114.239004 24820026 PMC4081338

[B7] Ball C. C. Parsons A. J. Rasmussen S. Shaw C. Rowarth J. S. (2012). Seasonal differences in the capacity of perennial ryegrass to respond to gibberellin explained. *Proc. N. Z. Grassland Assoc.* 74 183–188 10.1104/pp.114.239004

[B8] Benedict C. Skinner J. S. Meng R. Chang Y. Bhalereao R. Huner N. P. A. (2006). The CBF1-dependent low temperature signalling pathway, regulon and increase in freeze tolerance are conserved in *Populus* spp. *Plant Cell Environ.* 29 1259–1272 10.1111/j.1365-3040.2006.01505.x 17080948

[B9] Busov V. Meilan R. Pearce D. W. Rood S. B. Ma C. Tschaplinski T. J. (2006). Transgenic modification of *gai* or *rgl1* causes dwarfing and alters gibberellins, root growth, and metabolite profiles in *Populus*. *Planta* 224 288–296 10.1007/s00425-005-0213-9 16404575

[B10] Campbell C. Atkinson L. Zaragoza-Castells J. Lundmark M. Atkin O. Hurry V. (2007). Acclimation of photosynthesis and respiration is asynchronous in response to changes in temperature regardless of plant functional group. *New Phytol.* 176 375–389 10.1111/j.1469-8137.2007.02183.x 17692077

[B11] Claeys H. De Bodt S. Inzé D. (2014). Gibberellins and DELLAs: central nodes in growth regulatory networks. *Trends Plant Sci.* 19 231–239 10.1016/j.tplants.2013.10.001 24182663

[B12] Colebrook E. H. Thomas S. G. Phillips A. L. Hedden P. (2014). The role of gibberellin signalling in plant responses to abiotic stress. *J. Exp. Bot.* 217 67–75 10.1242/jeb.089938 24353205

[B13] Coleman H. D. Yan J. Mansfield S. D. (2009). Sucrose synthase affects carbon partitioning to increase cellulose production and altered cell wall structure. *Proc. Natl. Acad. Sci. U.S.A.* 106 13118–13123 10.1073/pnas.0900188106 19625620 PMC2722352

[B14] Cooke J. E. K. Eriksson M. E. Junttila O. (2012). The dynamic nature of bud dormancy in trees: environmental control and molecular mechanisms. *Plant Cell Environ.* 35 1707–1728 10.1111/j.1365-3040.2012.02552.x 22670814

[B15] Cosgrove D. J. Li C. L. Cho H.-T. Hoffmann-Benning S. Moore R. C. Blecker D. (2002). The growing world of expansins. *Plant Cell Physiol.* 43 1436–1444 10.1093/pcp/pcf180 12514240

[B16] Davies A. Evans M. E. Pollock C. J. (1989). Influence of date of tiller origin on leaf extension rates in perennial and Italian ryegrass at 15°C in relation to flowering propensity and carbohydrate status. *Ann. Bot.* 63 377–384.

[B17] Debast S. Nunes-Nesi A. Hajirezaei M. R. Hofmann J. Sonnewald U. Fernie A. R. (2011). Altering trehalose-6-phosphate content in transgenic potato tubers affects tuber growth and alters responsiveness to hormones during sprouting. *Plant Physiol.* 156 1754–1771 10.1104/pp.111.179903 21670224 PMC3149945

[B18] Eriksson M. E. Moritz T. (2002). Daylength and spatial expression of a gibberellin 20-oxidase isolated from hybrid aspen (*Populus tremula* L. *P. tremuloides Michx.). Planta* 214 920–930 10.1007/s00425-001-0703-3 11941469

[B19] Eriksson M. E. Webb A. A. R. (2011). Plant cell responses to cold are all about timing. *Curr. Opin. Plant Biol.* 14 731–737 10.1016/j.pbi.2011.08.005 21937261

[B20] Franklin K. A. Whitelam G. C. (2007). Light-quality regulation of freezing tolerance in *Arabidopsis thaliana*. *Nat. Gen.* 39 1410–1413 10.1038/ng.2007.3 17965713

[B21] Gilmour S. J. Sebolt A. M. Salazar M. P. Everard J. D. Thomashow M. F. (2000). Overexpression of the *Arabidopsis* CBF3 transcriptional activator mimics multiple biochemical changes associated with cold acclimation. *Plant Physiol.* 124 1854–1865 10.1104/pp.124.4.1854 11115899 PMC59880

[B22] Gorsuch P. A. Pandey S. Atkin O. K. (2010). Temporal heterogeneity of cold acclimation phenotypes in *Arabidopsis* leaves. *Plant Cell Environ.* 33 244–258 10.1111/j.1365-3040.2009.02074.x 19906148

[B23] Hacker J. Neuner G. (2006). Photosynthetic capacity and PSII efficiency of the evergreen alpine cushion plant *Saxifraga paniculata* during winter at different altitudes. *Arct. Antarct. Alp. Res.* 38 198–205 10.1657/1523-0430(2006)38[198:PCAPEO]2.0.CO;2

[B24] Heinrich M. Hettenhausen C. Lange T. Wünsche H. Fang J. J. Baldwin I. T. (2013). High levels of jasmonic acid antagonize the biosynthesis of gibberellins and inhibit the growth of *Nicotiana attenuata* stems. *Plant J.* 73 591–606 10.1111/tpj.12058 23190261

[B25] Heo J.-Y. Feng D. Niu X. Mitchell-Olds T. van Tienderen P. H. Tomes D. (2014). Identification of quantitative trait loci and a candidate locus for freezing tolerance in controlled and outdoor environments in the overwintering crucifer *Boechera stricta*. *Plant Cell Environ.* 37 2459–2469 10.1111/pce.12365 24811132 PMC4416058

[B26] Hoch B. Körner C. (2003). The carbon charging of pines at the climatic treeline: a global comparison. *Oecologia* 135 10–21 10.1007/s00442-002-1154-7 12647099

[B27] Hoch B. Körner C. (2009). Growth and carbon relations of tree line forming conifers at constant vs. variable low temperatures. *J. Ecol.* 97 57–66 10.1111/j.1365-2745.2008.01447.x

[B28] Holaday A. S. Martindale W. Alred R. Brooks A. L. Leegood R. C. (1992). Changes in activities of enzymes of carbon metabolism in leaves during exposure of plants to low temperature. *Plant Physiol.* 98 1105–1114 10.1104/pp.98.3.1105 16668733 PMC1080314

[B29] Hu Y. Jiang L. Wang F. Yu D. (2013). Jasmonate regulates the INDUCER OF CBF EXPRESSION-C-REPEAT BINDING FACTOR/DRE BINDING FACTOR1 cascade and freezing tolerance in *Arabidopsis*. *Plant Cell* 25 2907–2924 10.1105/tpc.113.112631 23933884 PMC3784588

[B30] Hüner N. P. A. Dahal K. Kurepin L. V. Savitch L. Singh J. Ivanov A. G. (2014). Potential for increased photosynthetic performance and crop productivity in response to climate change: role of CBFs and gibberellic acid. *Front. Chem.* 2:18 10.3389/fchem.2014.00018 PMC4029004 24860799

[B31] Hurry V. M. Strand Å. Tobiæson M. Gardeström P. Öquist G. (1995). Cold hardening of spring and winter wheat and rape results in differential effects on growth, carbon metabolism, and carbohydrate content. *Plant Physiol.* 109 697–706. 12228623 10.1104/pp.109.2.697 PMC157638

[B32] Kaplan F. Guy C. L. (2005). RNA interference of *Arabidopsis* beta-amylase8 prevents maltose accumulation upon cold shock and increases sensitivity of PSII photochemical efficiency to freezing stress. *Plant J.* 44 730–743 10.1111/j.1365-313X.2005.02565.x 16297066

[B33] Knight M. R. Knight H. (2012). Low-temperature perception leading to gene expression and cold tolerance in higher plants. *New Phytol.* 195 737–751 10.1111/j.1469-8137.2012.04239.x 22816520

[B34] Körner C. (2008). Winter crop growth at low temperature may hold the answer for alpine treeline formation. *Plant Ecol. Divers.* 1 3–11 10.1080/17550870802273411

[B35] Kurepin L. V. Dahal K. P. Savitch L. V. Singh J. Bode R. Ivanov A. G. (2013). Role of CBFs as integrators of chloroplast redox, phytochrome and plant hormone signaling during cold acclimation. *Int. J. Mol. Sci.* 14 12729–12763 10.3390/ijms140612729 23778089 PMC3709810

[B36] Lee C.-M. Thomashow M. F. (2012). Photoperiodic regulation of the C-repeat binding factor (CBF) cold acclimation pathway and freezing tolerance in *Arabidopsis thaliana*. *Proc. Natl. Acad. Sci. U.S.A.* 109 15054–15059 10.1073/pnas.1211295109 22927419 PMC3443188

[B37] Li T. A. Xu S. L. Oses-Prieto J. A. Putil S. Xu P. Wang R. J. (2011). Proteomics analysis reveals post-translational mechanisms for cold-induced metabolic changes in *Arabidopsis*. *Mol. Plant* 4 361–374 10.1093/mp/ssq078 21242321 PMC3063518

[B38] Liu Q. Kasuga M. Sakuma Y. Abe H. Miura S. Yamaguchi-Shinozaki K. (1998). Two transcription factors, DREB1 and DREB2 with an EREBP/AP2 DNA binding domain separate two cellular signal transduction pathways in drought- and low-temperature-responsive gene expression, respectively, in *Arabidopsis*. *Plant Cell* 8 1391–1406 10.2307/3870648 9707537 PMC144379

[B39] MacMillan C. P. Blundell C. A. King R. W. (2005). Flowering in the grass *Lolium perenne*. Effects of vernalization and long days on gibberellin biosynthesis and signaling. *Plant Physiol.* 138 1794–1806 10.1104/pp.105.062190 15980191 PMC1176447

[B40] Martin L. K. Haigler C. H. (2004). Cool temperature hinders flux from glucose to sucrose during cellulose synthesis in secondary wall stage cotton fibers. *Cellulose* 11 339–349 10.1023/B:CELL.0000046420.10403.15

[B41] Martínez-Barajas E. Delatte T. Schluepmann H. de Jong G. J. Somsen G. W. Nunes C. (2011). Wheat grain development is characterized by remarkable trehalose 6-phosphate accumulation pregrain filling: tissue distribution and relationship to SNF1-related protein kinase1 activity. *Plant Physiol.* 156 373–381 10.1104/pp.111.174524 21402798 PMC3091070

[B42] Martins M. C. Hejazi M. Fettke J. Steup M. Feil R. Krause U. (2013). Feedback inhibition of starch degradation in *Arabidopsis* leaves mediated by trehalose 6-phosphate. *Plant Physiol.* 163 1142–1163 10.1104/pp.113.226787 24043444 PMC3813640

[B43] Masclaux-Daubresse C. Purdy S. Lemaitre T. Pourtau N. Taconnat L. Renou J.-P. (2007). Genetic variation suggests interaction between cold acclimation and metabolic regulation of leaf senescence. *Plant Physiol.* 143 434–446 10.1104/pp.106.091355 17098848 PMC1761960

[B44] Molina-Montenegro M. A. Gallardo-Cerda J. Flores T. S. M. Atala C. (2012). The trade-off between cold resistance and growth determines the *Nothofagus pumilio* treeline. *Plant Ecol.* 213 133–142 10.1007/s11258-011-9964-5

[B45] Mølmann J. A. Asante D. K. A. Jensen J. B. Krane M. N. Ernstsen A. Junttila O. (2005). Low night temperature and inhibition of gibberellin biosynthesis override phytochrome action and induce bud set and cold acclimation, but not dormancy in PHYA overexpressors and wild-type of hybrid aspen. *Plant Cell Environ.* 28 1579–1588 10.1111/j.1365-3040.2005.01395.x

[B46] Mølmann J. A. Berhanu A. T. Stormo S. K. Ernstsen A. Junttila O. Olsen J. E. (2003). Metabolism of gibberellin A19 is under photoperiodic control in *Populus*, *Salix* and *Betula*, but not in daylength-sensitive *Populus* overexpressing phytochrome A. *Physiol. Plant.* 119 278–286 10.1034/j.1399-3054.2003.00176.x

[B47] Muller B. Pantin F. Génard M. Turc O. Freixes S. Piques M. (2011). Water deficits uncouple growth from photosynthesis, increase C content, and modify the relationship between C and growth in sink organs. *J. Exp. Bot.* 62 1715–1729 10.1093/jxb/erq438 21239376

[B48] Müller J. Aeschbacher R. A. Sprenger N. Boller T. Wiemken A. (2000). Disaccharide-mediated regulation of sucrose:fructan-6-fructosyltransferase, a key enzyme of fructan synthesis in barley leaves. *Plant Physiol.* 123 265–273 10.1104/pp.123.1.265 10806243 PMC59000

[B49] Noir S. Bömer M. Takahashi N. Ishida T. Tsui T.-L. Balbi V. (2013). Jasmonate controls leaf growth by repressing cell proliferation and the onset of endoreduplication while maintaining a potential stand-by mode. *Plant Physiol.* 161 1930–1951 10.1104/pp.113.214908 23439917 PMC3613466

[B50] Nunes C. O’Hara L. E. Primavesi L. F. Delatte T. L. Schluepmann H. Somsen G. (2013a). The trehalose 6-phosphate/SnRK1 signaling pathway primes growth recovery following relief of sink limitation. *Plant Physiol.* 162 1720–1732 10.1104/pp.113.220657 23735508 PMC3707538

[B51] Nunes C. Schluepmann H. Delatte T. L. Wingler A. Silva A. B. Feveiro P. S. (2013b). Regulation of growth by the trehalose pathway. Relationship to temperature and sucrose. *Plant Signal. Behav.* 8:e26626 10.4161/psb.26626 PMC4091418 24084646

[B52] O’Hara L. E. Paul M. J. Wingler A. (2013). How do sugars regulate plant growth and development? New insight into the role of trehalose-6-phosphate. *Mol. Plant* 6 261–273 10.1093/mp/sss120 23100484

[B53] Olsen J. E. (2010). Light and temperature sensing and signaling in induction of bud dormancy in woody plants. *Plant Mol. Biol.* 73 37–47 10.1007/s11103-010-9620-9 20213333

[B54] Öquist G. Huner N. P. A. (2003). Photosynthesis of overwintering evergreen plants. *Annu. Rev. Plant Biol.* 54 329–355 10.1146/annurev.arplant.54.072402.115741 14502994

[B55] Østrem L. Rapacz M. Jørgensen M. Höglind M. (2010). Impact of frost and plant age on compensatory growth in timothy and perennial ryegrass during winter. *Grass Forage Sci.* 65 15–22 10.1111/j.1365-2494.2009.00715.x

[B56] Pantin F. Simonneau T. Rolland G. Dauzat M. Muller B. (2011). Control of leaf expansion: a developmental switch from metabolics to hydraulics. *Plant Physiol.* 156 803–815 10.1104/pp.111.176289 21474437 PMC3177277

[B57] Park J.-Y. Canam T. Kang K.-Y. Unda F. Mansfield S. D. (2009). Sucrose phosphate synthase expression influences poplar phenology. *Tree Physiol.* 29 937–946 10.1093/treephys/tpp028 19429901

[B58] Parsons A. J. Rasmussen S. Liu Q. Xue H. Ball C. Shaw C. (2013). Plant growth – resource or strategy limited: insights from responses to gibberellin. *Grass Forage Sci.* 68 577–588 10.1111/gfs.12035

[B59] Parsons A. J. Robson M. R. (1980). Seasonal changes in the physiology of S24 perennial ryegrass (*Lolium perenne* L.). 1. Response of leaf extension to temperature during the transition from vegetative to reproductive growth. *Ann. Bot.* 46 435–444.

[B60] Paul M. Jhurreea D. Zhang Y. Primavesi L. Delatte T. Schluepmann H. (2010). Up-regulation of biosynthetic processes associated with growth by trehalose 6-phosphate. *Plant Signal. Behav.* 5 1–7 10.4161/psb.5.4.10792 20139731 PMC2958589

[B61] Paulsen J. Körner C. (2014). A climate-based model to predict potential treeline position around the globe. *Alp. Bot.* 124 1–12 10.1007/s00035-014-0124-0

[B62] Peacock J. M. (1975). Temperature and leaf growth in *Lolium perenne*. III. Factors affecting seasonal differences. *J. Appl. Biol.* 12 685–697 10.2307/2402182

[B63] Pollock C. J. Jones T. (1979). Seasonal patterns of fructan metabolism in forage grasses. *New Phytol.* 83 9–15 10.1111/j.1469-8137.1979.tb00720.x

[B64] Pourtau N. Jennings R. Pelzer E. Pallas J. Wingler A. (2006). Effect of sugar-induced senescence on gene expression and implications for the regulation of senescence in *Arabidopsis*. *Planta* 224 556–568 10.1007/s00425-006-0243-y 16514542

[B65] Renaut J. Lutts S. Hoffmann L. Hausman J.-F. (2004). Responses of poplar to chilling temperatures: proteomic and physiological aspects. *Plant Biol.* 6 81–90 10.1055/s-2004-815733 15095138

[B66] Reyez-Díaz M. Alberdi M. Piper F. Bravo L. A. Corcuera L. J. (2005). Low temperature responses to *Nothofagus dombeyi* and *Nothofagus nitida*, two evergreen species from south central Chile. *Tree Physiol.* 25 1389–1398 10.1093/treephys/25.11.1389 16105806

[B67] Rinne P. Welling W. Kaikuranta P. (1998). Onset of freezing tolerance in birch (*Betula pubescencs* Erh.) involves LEA proteins and osmoregulation and is impaired in and ABA-deficient genotype. *Plant Cell Environ.* 21 601–611 10.1046/j.1365-3040.1998.00306.x

[B68] Rohde A. Prinsen E. De Rycke R. Engler G. Van Montagu M. Boerjan W. (2002). PtABI3 impinges on the growth and differentiation of embryonic leaves during bud set in poplar. *Plant Cell* 14 1885–1901 10.1105/tpc.003186 12172029 PMC151472

[B69] Rolland F. Moore B. Sheen J. (2002). Sugar sensing and signaling in plants. *Plant Cell* 14(Suppl. 1), S185–S205 10.1105/tpc.010455 12045277 PMC151255

[B70] Sandve S. R. Kosmala A. Rudi H. Fjellheim S. Rapacz M. Yamada T. (2011). Molecular mechanisms underlying frost tolerance in perennial grasses adapted to cold climates. *Plant Sci.* 180 69–77 10.1016/j.plantsci.2010.07.011 21421349

[B71] Savitch L. V. Barker-Åstrom J. Invanov A. G. Hurry V. Öquist G. Huner N. P. A. (2001). Cold acclimation of *Arabidopsis* thaliana results in incomplete recovery of photosynthetic capacity, associated with an increased reduction of the chloroplast stroma. *Planta* 214 295–303 10.1007/s004250100622 11800395

[B72] Savitch L. V. Harney T. Huner N. P. A. (2000). Sucrose metabolism in spring and winter wheat in response to high irradiance, cold stress and cold acclimation. *Physiol. Plant.* 108 270–278 10.1034/j.1399-3054.2000.108003270.x

[B73] Schluepmann H. Berke L. Sanchez-Perez G. F. (2012). Metabolism control over growth: a case for trehalose-6-phosphate in plants. *J. Exp. Bot.* 63 3379–3390 10.1093/jxb/err311 22058405

[B74] Seo E. Lee H. Jeon J. Park H. Kim J. Noh Y.-S. (2009). Crosstalk between cold response and flowering in *Arabidopsis* is mediated through the flowering-time gene SOC1 and its upstream negative regulator FLC. *Plant Cell* 21 3185–3197 10.1105/tpc.108.063883 19825833 PMC2782271

[B75] Sharma P. Sharma N. Deswal R. (2005). The molecular biology of the low-temperature response in plants. *Bioessays* 27 1048–1059 10.1002/bies.20307 16163711

[B76] Shi P. Körner C. Hoch G. (2008). A test of the growth-limitation theory for alpine tree line formation in evergreen and deciduous taxa of the eastern Himalayas. *Funct. Ecol.* 22 213–220 10.1111/j.1365-2435.2007.01370.x

[B77] Skinner H. (2007). Winter carbon dioxide fluxes in humid-temperate pastures. *Agric. Forest Meteorol.* 144 32–43 10.1016/j.agrformet.2007.01.010

[B78] Skirycz A. Inzé D. (2010). More from less: plant growth under limited water. *Curr. Opin. Plant Biol.* 21 197–203 10.1016/j.copbio.2010.03.002 20363612

[B79] Song S. Qi T. Wasternack C. Xie D. (2014). Jasmonate signaling and crosstalk with gibberellin and ethylene. *Curr. Opin. Plant Biol.* 21 112–119 10.1016/j.pbi.2014.07.005 25064075

[B80] Stapleton J. Jones M. B. (1987). Effects of vernalization on the subsequent rates of leaf extension and photosynthesis of perennial ryegrass (*Lolium perenne* L.). *Grass Forage Sci.* 47 27–31 10.1111/j.1365-2494.1987.tb02087.x

[B81] Strand Å. Foyer C. H. Gustafsson P. Gardeström P. Hurry V. (2003). Altering flux through the sucrose biosynthesis pathway in transgenic *Arabidopsis* thaliana modifies photosynthetic acclimation at low temperatures and the development of freezing tolerance. *Plant Cell Environ.* 26 523–535 10.1046/j.1365-3040.2003.00983.x

[B82] Strand Å. Hurry V. Gustafsson P. Gardeström P. (1997). Development of *Arabidopsis* thaliana leaves at low temperatures releases the suppression of photosynthesis and photosynthetic gene expression despite the accumulation of soluble carbohydrates. *Plant J.* 12 605–614 10.1046/j.1365-313X.1997.00605.x 9351245

[B83] Strand Å. Hurry V. Henkes S. Huner N. Gustafsson P. Gardeström P. (1999). Acclimation of *Arabidopsis* leaves developing at low temperatures. Increasing cytoplasmic volume accompanies increased activities of enzymes in the Calvin Cycle and in the sucrose-biosynthesis pathway. *Plant Physiol.* 119 1387–1397 10.1104/pp.119.4.1387 10198098 PMC32024

[B84] Sulpice R. Pyl E.-T. Ishihara H. Trenkamp S. Steinfath M. Witucka-Wall H. (2009). Starch as a major integrator in the regulation of plant growth. *Proc. Natl. Acad. Sci. U.S.A.* 106 10348–10353 10.1073/pnas.0903478106 19506259 PMC2693182

[B85] Tamura K. Sanada Y. Tase K. Yoshida M. (2014). Fructan metabolism and expression of genes coding fructan metabolic enzymes during cold acclimation and overwintering in timothy (*Phleum pratense*). *J. Plant Physiol.* 171 951–958 10.1016/j.jplph.2014.02.007 24913052

[B86] Tardieu F. Parent B. Simonneau T. (2010). Control of leaf growth by abscisic acid: hydraulic or non-hydraulic processes? *Plant Cell Environ.* 33 636–647 10.1111/j.1365-3040.2009.02091.x 20002334

[B87] Tinus R. W. Burr K. E. Atzmon N. Riov J. (2000). Relationship between carbohydrate concentration and root growth potential in coniferous seedlings from three climates during cold hardening and dehardening. *Tree Physiol.* 20 1097–1104 10.1093/treephys/20.16.1097 11269961

[B88] Van den Ende W. (2013). Multifunctional fructans and raffinose family oligosaccharides. *Front. Plant Sci.* 4:247 10.3389/fpls.2013.00247 PMC3713406 23882273

[B89] Van den Ende W. De Coninck B. Van Laere A. (2004). Plant fructan exohydrolase: a role in signaling and defense? *Trends Plant Sci*. 9 523–528 10.1016/j.tplants.2004.09.008 15501176

[B90] Wasternack C. (2014). Action of jasmonates in plant stress responses and development – applied aspects. *Biotechnol. Adv.* 32 31–39 10.1016/j.biotechadv.2013.09.009 24095665

[B91] Welling A. Kaikuranta P. Rinne P. (1997). Photoperiodic induction of dormancy and freezing tolerance in *Betula pubescens*. Involvement of ABA and dehydrins. *Physiol. Plant.* 100 119–125 10.1034/j.1399-3054.1997.1000112.x

[B92] Welling A. Moritz T. Palva E. T. Junttila O. (2002). Independent activation of cold acclimation by low temperature and short photoperiod in hybrid aspen. *Plant Physiol.* 129 1633–1641 10.1104/pp.003814 12177476 PMC166751

[B93] Welling A. Palva E. T. (2008). Involvement of CBF transcription factors in winter hardiness in birch. *Plant Physiol.* 147 1199–1211 10.1104/pp.108.117812 18467468 PMC2442524

[B94] Wild M. Davière J.-M. Cheminant S. Regnault T. Baumberger N. Heintz D. (2012). The *Arabidopsis* DELLA RGA-LIKE3 is a direct target of MYC2 and modulates jasmonate signaling responses. *Plant Cell* 24 3307–3319 10.1105/tpc.112.101428 22892320 PMC3462633

[B95] Wingler A. (2011). Interactions between flowering and senescence regulation and the influence of low temperature in *Arabidopsis* and crop plants. *Ann. Appl. Biol.* 159 320–338 10.1111/j.1744-7348.2011.00497.x

[B96] Wingler A. Juvany M. Cuthbert C. Munné-Bosch S. (2014). Adaptation to altitude affects the senescence response to chilling in the perennial plant *Arabis alpina*. *J. Exp. Bot.* 10.1093/jxb/eru426 [Epub ahead of print]. PMC4265169 25371506

[B97] Wingler A. Masclaux-Daubresse C. Fischer A. M. (2009). Sugars, senescence and ageing in plants and heterotrophic organisms. *J. Exp. Bot.* 60 1063–1066 10.1093/jxb/erp067 19276191

[B98] Wingler A. Stangberg E. J. Saxena T. Mistry R. (2012). Interactions between temperature and sugars in the regulation of leaf senescence in the perennial herb *Arabis alpina* L. *J. Integr. Plant Biol.* 54 595–605 10.1111/j.1744-7909.2012.01145.x 22788771

[B99] Xiong Y. Fei S.-Z. (2006). Functional and phylogenetic analysis of a DREB/CBF-like gene in perennial ryegrass (*Lolium perenne* L.). *Planta* 224 878–888 10.1007/s00425-006-0273-5 16614820

[B100] Xu H. Liu Q. Yao T. Fu X. (2014). Shedding light on integrative GA signaling. *Curr. Opin. Plant Biol.* 21 89–95 10.1016/j.pbi.2014.06.010 25061896

[B101] Yadav U. P. Ivakov A. Feil R. Duan G. Y. Walther D. Giavalisco P. (2014). The sucrose-trehalose 6-phosphate (Tre6P) nexus: specificity and mechanisms of sucrose signalling by Tre6P. *J. Exp. Bot.* 65 1051–1068 10.1093/jxb/ert457 24420566 PMC3935566

[B102] Yano R. Nakamura M. Yoneyama T. Nishida I. (2005). Starch-related α-glucan/water dikinase is involved in the cold-induced development of freezing tolerance in *Arabidopsis*. *Plant Physiol.* 138 837–846 10.1104/pp.104.056374 15894744 PMC1150401

[B103] Zawaski C. Busov V. B. (2014). Roles of gibberellin catabolism and signaling in growth and physiological responses to drought and short-day photoperiods in *Populus* trees. *PLoS ONE* 9:e86217 10.1371/journal.pone.0086217 PMC3896445 24465967

[B104] Zawaski C. Kadmiel M. Pickens J. Ma C. Strauss S. Busov V. (2011). Repression of gibberellin biosynthesis or signaling produces striking alterations in poplar growth, morphology, and flowering. *Planta* 234 1285–1298 10.1007/s00425-011-1485-x 21792553

[B105] Zhang Y. Primavesi L. F. Jhurreea D. Andralojc P. J. Mitchell R. A. Powers S. J. (2009). Inhibition of Snf1-related protein kinase (SnRK1) activity and regulation of metabolic pathways by trehalose 6-phosphate. *Plant Physiol.* 149 1860–1871 10.1104/pp.108.133934 19193861 PMC2663748

[B106] Zheng M. Wang Y. Liu K. Shu H. Zhou Z. (2012). Protein expression changes during cotton fiber elongation in response to low temperature stress. *J. Plant Physiol.* 169 399–409 10.1016/j.jplph.2011.09.014 22244703

[B107] Zhou M. Xu M. Wu L. Shen C. Ma H. Lin J. (2014). CbCBF from *Capsella bursa-pastoris* enhances cold tolerance and restrains growth in *Nicotiana tabacum* by antagonizing with gibberellin and affecting cell cycle signaling. *Plant Mol. Biol.* 85 259–275 10.1007/s11103-014-0181-1 24532380

